# Actively Addressing Systemic Racism Using a Behavioral Community Approach

**DOI:** 10.1007/s42822-022-00101-6

**Published:** 2022-10-17

**Authors:** Jomella Watson-Thompson, Ruaa H. Hassaballa, Stephanie H. Valentini, Jonathan A. Schulz, Priya Vanchy Kadavasal, Joshua D. Harsin, Valerie M. Thompson, Ithar H. Hassaballa, Cynthia C. Esiaka, Eric C. Thompson

**Affiliations:** 1 Department of Applied Behavioral Science, University of Kansas, 1000 Sunnyside Avenue, Room 4001, Lawrence, KS 66045 USA; 2 School of Education and Human Sciences, University of Kansas, Lawrence, KS USA

**Keywords:** Systemic racism, Ally, Behavioral community, Ecological

## Abstract

Recent police brutality and related violence against Black people, coupled with the COVID-19 pandemic, has further evidenced the disproportionate impact of systemic racism in our institutions and across society. In the United States, the alarming mortality rates for Black people due to police violence and COVID-19 related deaths are clear demonstrations of inequities within a long history of disparate outcomes. In understanding systemic racism, it is essential to consider how it is embedded within society and across socio-ecological levels. The Social-Ecological Model (SEM) is used to examine conditions within the environment that maintain systemic racism, including within our field and discipline. A behavioral-community approach for examining racism aids in determining points of intervention across multiple ecological levels that may contribute to behavior change, including with behaviorists. The science of behavior is well-suited to help examine the contingencies governing behaviors within and across systems, which is pivotal for addressing operant behaviors to influence long-term behavior change. This paper calls on the behavioral community to address systemic racism within our environments and systems of influence to contribute to a more equitable community. Systemic racism, including within the context of anti-Blackness, is examined by considering behavior change strategies that can be supported by behaviorists across socio-ecological levels. Tools for collaborative action are provided to support behaviorists in demonstrating the skills needed across a continuum of behaviors from allyship to anti-racism to actively address systemic racism.

If you are neutral in situations of injustice, you have chosen the side of the oppressor.

—Archbishop Desmond Tutu, *Attributed 1986*

Recent police brutality and related violence against Black people, coupled with the COVID-19 pandemic, further evidenced the disproportionate impact of systemic racism in our institutions and across society. The alarming rates by which Black people are experiencing deaths related to COVID-19 and police-related violence are more recent demonstrations of inequities within a long history of disparate outcomes (Iacobucci, 2020; Pachter & Coll, 2009). In the United States, Black people are killed by the police (Sinyangwe et al., 2020) and have experienced COVID-19 related deaths at nearly three times the rate of White people (Marchildon, 2020). The immediate and pressing concerns experienced by Black people in the United States are notably manifested in health inequities (e.g., COVID-19 exposure and deaths) and social injustices (e.g., police brutality).

In the days of unrest following the May 25, 2020, murder of George Floyd, people shared reading lists on racism, urged others to examine their privilege, and called for monetary donations and crowdfunding to begin to mobilize for action. While all these are important and meaningful steps, it is necessary to continue to probe more deeply regarding how to address the range of inequities, which collectively contribute to the disparate outcomes, particularly as experienced by Black people in the United States. This paper explores how to address systemic racism overall and within the context of anti-Blackness, through active behaviors that may contribute to a more just and equitable community.

## Anti-Black Racism

In the United States, systemic racism is a manifestation of a much deeper issue including anti-Black racism, which “is a specific form of racism, rooted in the history and experience of enslavement, that is targeted against Black people” (Dryden & Nnonrom, 2021). To continue to ignore anti-Black racism within the broader context of systemic racism inhibits our ability to more effectively support actions to meaningfully address the specific issue. At times, taking a universal, or one-size fits all approach to addressing racism, may be harmful or even damaging for advancing systemic and sustainable change, particularly within the context of anti-Black racism in the United States.

In general, individuals and groups of different races and ethnicities may be considered members of a stimulus class that shares similar physical (e.g., skin tone, facial features), temporal (e.g., genetic ancestry), or functional (i.e., privilege, advantage/disadvantage) dimensions (Cooper et al., 2007). Often, the response topography or form of the behavior (e.g., pulled over by an officer, followed in a store) in response to a member of the stimulus class (e.g., Black, Indigenous, & People of Color or BIPOC) may seem to have similar topographical components based on what the behavior looks like, but there are often variances (e.g., differential treatment of an individual when stopped by police or followed in store). The magnitude of the behavioral response, including the intensity, force, and severity, may often be different based on the stimulus (e.g., physical attribute of person). Thus, it is critical to examine the magnitude and the function of the behavior. Additionally, the function or consequence of the behavior also should be examined to better understand what may be part of a response class or group of responses that serve the same or different function based on the members (e.g., Black individuals) of a stimulus class (e.g., BIPOC). It is important to analyze racist behaviors and the specific forms (e.g., anti-Black racism) to understand what may be the most appropriate active behaviors or responses.

From a behavioral perspective, anti-Black actions are setting events that provide a historical context for the specific form of racism. Anti-Blackness may be defined as,

actions or behaviors that minimize, marginalize, or devalue the full participation of Black people in life. The spectrum of anti-Black actions and behaviors span from unconscious bias to motivated acts of prejudice. They include the tolerance of or indifference to the under-representation, differential success and advance, or experience of Black people. (University of California Irvine, 2020, “Anti-Blackness: A Definition”)

Unconscious or implicit bias can be defined as “behavior that is influenced in an implicit manner by cues that function as an indicator of the social group to which others belong…The influence of these social cues can be labeled as implicit when it occurs quickly, effortlessly…or in a way that is difficult to control” (De Houwer, 2019, p. 836). Implicit bias may contribute to racial resentment, including stereotypes in which attitudes and perceptions about specific characteristics (e.g., physical attributes) or behaviors (e.g., work style) are generalized to all members of a group. Whereas explicit bias is overt behaviors of prejudice and discrimination that is often observable through verbal behavior (i.e., speech), physical expression (e.g., gestures), or other actions (Man, 2021). Through responses that may be either overt (i.e., active racism) or covert (i.e., passive racism), anti-Black racism is experienced at the individual (e.g., personal), group (i.e., interpersonal, organizational), and societal levels including through policies, institutional practices, and norms that often were historically and intentionally anti-Black (e.g., segregation; redlining; hiring practices) (Man, 2021).

## Continuum of Active Behaviors to Disrupt Racism

**Fig. 1:**
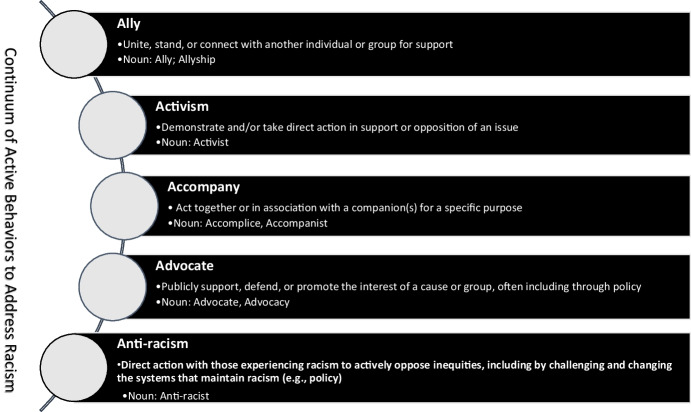
Continuum of active behaviors to address racism

A continuum of active behaviors that can be supported by individuals and/or groups to better understand and respond to racism is presented in Fig. 1 and includes ally, accompany, advocate, activism, and anti-racism. Several of the active behaviors may be considered different components of activism including to accompany, advocate, and anti-racism (Ardila-Sanchez et al., 2020). As such, it is important to note that the behaviors presented across the continuum are interactive, dynamic, and often integrated. The continuum presents the active behaviors as actions (e.g., activism) to be supported rather than as a role (i.e., activist) to be achieved. Thus, the individuals or groups experiencing racism should be positioned to guide the more privileged person(s) offering support in determining the most appropriate form of action for the situation and based on the context. Additionally, the more privileged person(s) should avoid self-proclamation of their role (e.g., activist) and allow those served or experiencing the racism to determine whether to assign a title to the person.

Some considerations may guide the selection of appropriate actions and are worth noting to avoid disingenuity. A tension that may persist is based on the “ally industrial complex” in which the careers of individuals who serve as allies are interdependent on the issues they are addressing (El-Mekki, 2018). In this case, the action (e.g., allyship, activism) may be seen as tokenism or saviorism if those who are championing are primarily serving in this role (as allies or activists) for individuals with whom they have a direct relationship. To avoid ally industrial complex, it may be appropriate to be in accompaniment as an accomplice with the individuals and groups served by offering a range of support and direct actions. Additionally, it is important to avoid behaviors that are performative (e.g., performative allyship), in which individuals or groups may be serving for self-benefit or recognition based on what may be promoted as socially appropriate behavior (Atcheson, 2021). In this regard, maintaining a social justice focus (e.g., such as through social justice allyship) is critical to address the social issue, including racism, through collaborative partnerships to occasion individual (e.g., self-reflection) and collective accountability in dismantling systems (Edwards, 2006). In determining what may be the most appropriate action, a continuum of active behaviors should be considered with those most impacted and affected by racism to inform what may be the most helpful by those offering support based on the situation, history, and context (El-Mekki, 2018).

## Purpose of this Paper

This paper examines systemic racism, within the context of anti-Blackness, and also explores the importance of behaviorists in contributing to dismantling the problem within our own structures. It may be helpful to note that we are supporting a non-linear approach (e.g., Goldiamond, 1974; Layng et al., 2021) to behavior and systems change as it is important to examine both the absence (e.g., not providing equitable opportunities) and presence (e.g., occassioning equitable opportunities) of behaviors that one can engage in as anti-racist. Based on the earlier work of Goldiamond (1974), Layng et al. (2021) proposed nonlinear contingency analysis as a constructional approach that can aid individuals in identifying and establishing repretiores and patterns of behavior for which the absence of the behavior is otherwise a problem (e.g., not providing equitable opportunites).

The focus of this paper is to aid members of the behavioral community in addressing racism and anti-racist behaviors, including in identifying and modifiying our own behaviors and practices within and across our institutions and groups. Ideas for interventions that behaviorists can take within the environments we operate and have influence are discussed. Tools or strategies are presented that facilitate both individual and collaborative action to offer appropriate supports to actively demonstrate the empathy and skills needed.

The behaviors of those who may serve in the change process as targets (i.e., those whose behavior needs to change) and agents (i.e., those whose can contribute to facilitating change in the environment) are identified in this paper to help identify a range of behavior change strategies across a continumm of active behaviors that can be supported by individuals and groups. As we consider behavior change strategies, individuals or groups who may be the target of change may also serve as an agent or actor to facilitate the change activity (e.g., policy).

The authors of this manuscript identify across diverse racial and ethnic groups, with half of the team identifying as a Black person. In this paper, behaviorists will be the term used to broadly reference those who practice, research, or promote the science of behavior, including applied behavior analysts and applied behavioral scientists. Across the team, the authors professionally identify as applied behavior analysts, applied behavior scientists, and behavioral community psychologists. Based on the views and experiences of the author team, there is a continuum of supportive actions that may be appropriate in addressing systemic racism, including allyship, activism, accompaniment, advocacy, and anti-racism. In this paper, we challenge ourselves and other behaviorists to consider what may be active behaviors to support in addressing systemic and anti-Black racism. Thus, even in writing this manuscript the authors are individually and collecting examining our own behaviors in addressing anti-racism across settings and groups in which we may participate or have influence. Data sharing was not applicable to this article as no datasets were generated or analyzed during the current study.

## Systemic Racism and Social Determinants of Health

Systemic racism describes a societal structure that maintains a racially oppressive system that provides privileges to certain racial groups while oppressing others (Feagin, 2006). Although the terms are often used interchangeably, systemic racism is a form and product of structural racism. Structural racism is broader and includes the history, culture, and structure of the socioeconomic and political systems that enable racial inequity, including policies, norms, and institutional practices (Aspen Institute, 2016). Thus, systemic racism is embedded within and across systems of society and is a root cause of many disproportionate outcomes faced by Black people across education, economic, health, housing, and criminal justice systems (Prather et al., 2016; Trent et al., 2019). As such, more recently racism has been identified as an ongoing public health crisis affecting the overall health and well-being of all Americans (American Public Health Association, 2020).

Many of the factors influencing systemic racism are considered social determinants of health (SDOH), or underlying conditions in the environment in which people live that determine health and well-being (American Public Health Association, 2020; World Health Organization, 2008). Examples of SDOH include employment, social connections, and access to resources. Based on the SDOH, it is critical to consider the interactions between structural (e.g., policy, cultural norms, governance) and individual level (e.g., knowledge, skills) factors influencing inequities, including systemic racism. Structural mechanisms that influence social determinants include the socioeconomic and political context (e.g., policy, cultural norms, governance) of systems (e.g., health, justice), which then impact individual (e.g., educational level/knowledge), relational (e.g., social connectedness), and community (e.g., employment practices, media) level factors (Centers for Disease Control and Prevention, 2020). The present paper focuses on examining systemic racism and related factors influenced by the determinants of health.

Since racism is experienced across levels from the individual to the systemic levels, an ecological approach may aid in understanding and addressing the issue. Individual-level racism is a learned behavior. For this reason, anti-racist behavior can be learned and lived, as demonstrated by those who have actively challenged racism throughout history (Derman-Sparks & Phillips, 1997). It is key that behaviorists understand the broader context in which we do our work, beginning with an examination of our own experiences with and contributions to systemic racism.

## Behavioral Community Perspective: Broadening the Scope of Applied Behavior Analysis

Since systemic racism is embedded within and across structures of society, it is critical to examine the impact across the multiple aspects of the environment in which individuals and groups, including behaviorists, interact and behave (Watson-Thompson et al., 2020). Behavioral community psychology integrates both a behavior-analytic and community psychology perspective, which may offer a comprehensive approach for examining systemic racism through behavior change across multiple aspects of the environment. A behavioral community perspective occasions the opportunity to focus our attention, as behaviorists, on the setting events (e.g., history, experience) as the context and broader conditions (e.g., social justice, empowerment, inclusion) under which we advance our work (Bogat & Jason, 2000; Watson-Thompson et al., 2020). Through a behavioral community approach, a couple of core principles promoted include: (1) social justice to ensure equitable conditions and outcomes; and (2) equitable participation, including by those most affected by the issue, to lead the problem-solving. For instance, in supporting social justice, behaviorists may ensure access to resources, opportunities, and privileges with and for those who may be most underrepresented and disenfranchised within their systems (e.g., employees, members) and with whom they serve (e.g., access to services).

## Behaviorists’ Role in Addressing Systemic Racism

Behaviorists need to understand the systems in which we live and work, as well as how our individual and collective actions either prevent or contribute to systemic racism. In recognizing one’s position of power and privilege, it is important to consider how systemic racism is perpetuated structurally; thereby requiring behaviorists, even when engaged in providing services to underserved groups, to be accountable for how we operate within inherently racist systems. Behaviorists have the opportunity to intervene by implementing active behaviors to address racism across multiple levels (e.g., individual, interpersonal, community, societal) in which it may be ingrained in order to facilitate more equitable conditions. Whether working in practice, research, and/or academic settings, behaviorists should critically examine and actively contribute to addressing systemic racism in the environments in which they have influence (e.g., organizations, professional associations, workplaces, homes, universities). To partake in active behaviors addressing racial disparities and inequities, behaviorists should ensure their work is inclusive and participatory. For instance, when we critically identify and promote the advancement of Black behaviorists, then diverse perspectives are more readily apparent, authentic community representation is assured, and the likelihood of serving those most excluded is enhanced (Elias et al., 2015).

## Using the Social-Ecological Model to Address Systemic Racism

Behaviorists can work to dismantle the interrelated conditions of racism through systematic facilitation of strategies across ecological levels. To engage in active behaviors occassions considering how to promote equity across multiple levels, including at the individual, interpersonal, community, and societal levels. The socioecological model has been used by the CDC to examine the multiple and interrelated factors influencing violence across ecological levels. The Centers for Disease Control and Prevention’s (CDC) (2020) has promoted the Social-Ecological Model (SEM), derived from Bronfenbrenner’s (1979) approach, to understand influences that impact behavior across different parts of the environment or social ecology. Originally, Bronfenbrenner’s (1979) model examined ecological systems within the context of understanding child development based on influences across multiple environments and systems.

**Fig. 2:**
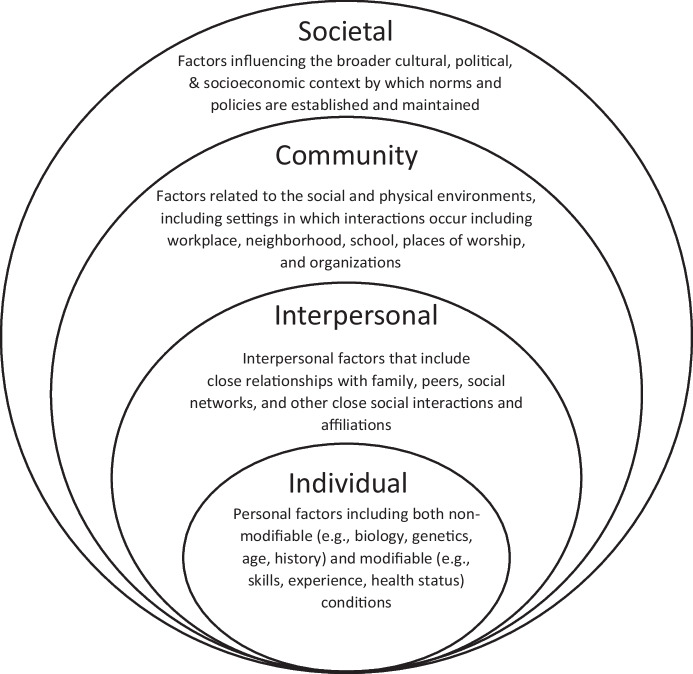
Socio-ecological levels adapted from the framework for prevention (Dahlberg & Krug, 2002)

The socioecological model is starting to be applied to examine racism and anti-racism (Harper Browne & O’Connor, 2021; Yulianto, 2020). To understand the influences and opportunities to address systemic racism, the SEM examines multiple levels including the: (a) Individual (i.e., Intrapersonal), (b) Relationship (i.e., Interpersonal), (c) Community, and (d) Societal. Figure 2 displays the different levels of the social-ecological model. The social-ecological perspective illustrates how factors at one level (e.g., policy) may impact other (e.g., community, individual) levels when addressing racism.

Additionally, the SEM offers a comprehensive approach to examine and address systemic racism by considering the multiple environments and points of influence in which individuals and groups behave. Using the SEM as a framework, a set of intervention components are presented that can be supported at each level of the ecological model. Behavior change strategies are implemented based on whose behavior should be modified, which includes identifying both the target and/or agents of change. Targets of change directly experience, contribute to, or are harmed by the problem of racism, in this case. Agents of change include those who can influence positive change in the environment, subsequently influencing others’ behavior. Those who serve as agents of change can also be the targets of change too if they are engaged in behaviors that may perpetuate or support racist actions through their verbal or physical behaviors or lack thereof. For instance, addressing racism in the workplace may first require that organizational leaders be targets of change and participate in self-reflection and awareness-building activities to identify their own biases that impact organizational decisions. Then, organizational leaders may later be able to serve as agents of change in developing and enforcing more equitable workplace policies that promote diversity and inclusion. In Table 1, intervention components are aligned by behavior change strategies (Community Toolbox, 2020a), which range from antecedent (e.g., providing information) to consequent (e.g., changing policy) interventions (Watson-Thompson et al., 2020).

**Table 1:** Behavior change strategies to address racism across ecological levels

Behavior Change Strategies	Illustrative Examples Across Ecological Levels of Actions to Support Active Behaviors
*Providing Information and Enhancing Skills* *Refers to occasioning knowledge and skill acquisition, including through antecedent conditions and establishing paired and conditioned reinforcement relations*	• Understand and identify areas of white fragility that may result in avoidance or punishment if addressing race-related issues (*Individual; Target)* • Review resources and increase understanding of microaggressions and macroaggressions related to racism in the workplace (*Individual; Target)* • Learn about the history and cultural context of Black people in the United States to increase cultural knowledge and humility; Learn about racism to not unnecessarily burden behavior analysts who are Black (*Interpersonal; Target)* • Provide opportunities to obtain information from those served regarding how to support culturally appropriate services (*Interpersonal; Target)* • Educate and provide feedback when observing instances of prejudice, discrimination, or racism with colleagues or those served (*Interpersonal; Agent)* • Incorporate diverse representation in curricula and training materials to be more representative of a broader population (*Community; Target)* • Promote organizational-wide behavioral skills training to increase cultural sensitivity (*Community; Agent)* • Require cultural competence and humility training and technical supports within educational and work settings (*Community; Agent)* • Provide expert statements and testimonies to advocate for the equitable delivery of services and/or treatment of populations served who may experience racism or discrimination (e.g., arrests of Black males with autism; insurance providers supporting ABA treatment) (*Societal; Agent)*
*Enhancing Services and Supports* *Refers to occasioning responding through changes in environmental variables and contingencies to support more equitable provision of services*	• Recognize and address the position of power (e.g., education, money, status) and privilege (e.g., tenure) that may influence service provision (*Individual; Target)* • Support self-assessment and reflection to identify biases that may influence service delivery (e.g., Project Implicit Assessment to examine biases) (*Individual; Target)* • Promote authentic relationships with others, including diverse colleagues and/or those served, to support equitable conditions as equal partners (e.g., clients, caregivers, co-workers) and reduce hierarchical relationships and power differentials (*Individual; Target)* • Actively listen to others from diverse backgrounds to better understand the history and experiences of colleagues within the field and/or with service providers, including behavior analysts (*Interpersonal; Target)* • Examine and promote the delivery of resources and services to diverse individuals and groups across demographic and geographical areas (C*ommunity; Agent)* • Ensure diversity of representation and inclusion in leadership and staffing structures of organizations and service providers (*Community; Target)* • Expand the range of areas in which ABA is supported (e.g., interdisciplinary) and promoted (e.g., publication) to address a range of issues (e.g., racism, behavior skills, and discrimination/bias training for law enforcement) (*Community; Target)*
*Modifying Access, Barriers, and Opportunities* *Refers to modification of functional relations between variables through differential reinforcement*	• Occasion the opportunities for others to be heard through situational assessment of whose voice is necessary and/or missing based on the settings and conditions (e.g., white-dominated space) (*Individual; Target)* • Provide opportunities for colleagues to connect and develop a community of support to engage in collective action (*Interpersonal; Target)* • Mentor and support students and colleagues of color to enhance networks and occasion opportunities for access to settings and support (*Interpersonal; Agent)* • Support and actively participate in groups (e.g., special interest groups) that promote interactions with diverse individuals and groups (*Interpersonal, Target)* • Ensure career opportunities are advertised in diverse spaces (*Community, Agent)* • Increase access to supervision in diverse settings and behaviors by connecting Black behavior analysts (e.g., professional development) (*Interpersonal; Agent)* • Compensate Black behavior analysts for their equity work as an area of expertise (*Community, Target)* • Conduct a cultural audit of work settings (i.e., who is delivering services) and service delivery (i.e., who is receiving services) (*Community, Target)* • Establish editorial policies to report on the demographic and geographic characteristics of participants (e.g., Black participants) and settings (e.g., urban schools) to understand the generality of findings for racial and ethnic groups (*Community; Target)* • Examine access in the provision and delivery of services to racial and ethnic groups (e.g., geography, demographics) (*Community; Target)* • Advocate for behavior analysts’ involvement in emergency response interventions with police (e.g., assisting officers when responding to calls) (*Societal; Agent)*
*Changing Consequences* *Refers to altering motivating and establishing operations that establish or alter the likelihood of a behavior or effectiveness of a consequence (e.g., incentives, disincentives)*	• Provide and incentivize opportunities to include colleagues, parents, and other people of color in dialogue and action for decision-making (*Community level; Target of change)* • Disarm microaggressions by calling attention to instances of racist behavior – name the problem and shift responsibility for addressing the problem (*Interpersonal; Agent)* • Provide incentives (e.g., membership, employment, recruitment) to increase participation of non-Black people in training and representation of Black people in the workforce (Community*; Agent)* • Engage external consultants in facilitating dialogues and activities within work settings and with those served to obtain feedback on diversity and inclusion (*Interpersonal; Agent)* • Provide paid absences for employee identified cultural and religious holidays (e.g., Juneteenth, Kwanzaa) (*Community; Target)* • Examine and address pay inequities that may disproportionately affect individuals who are BIPOC by promoting salary transparency and plans to address salary inequities (*Community; Target)* • Promote and ensure equitable representation in leadership and pay structures to attract and maintain service providers who identify as BIPOC (*Community; Target)*
*Modifying Policy and Broader Systems* *Refers to establishing consistent and predictable consequences; Increasing the prevalence of rule-governed behavior; Establishing cultural norms*	• Examine workplace policies to ensure procedures are in place that promote equitable treatment of employees and allow for all voices to be heard (*Community; Target)* • Examine how research is considered socially valid and the process by which it may diminish or limit research about Black communities (*Community; Agent)* • Establish policies to support diversity-related training and continuing education for all staff (*Community; Target)* • Modify policies that disproportionately impact Black people in the workplace or through service provision (e.g., bans on dress code, hairstyles) (*Community; Target)* • Advocate for the equitable delivery of services and/or treatment of populations served through examining pay mechanisms (e.g., insurance coverage) that may limit access to services (e.g., Black people with autism) (*Societal; Agent)* • Support reparations for Black people (e.g., educational scholarships, policies for equitable pay structures, supporting diversity suppliers) to address inequities in education and economics through restitutions (*Societal; Target*)

The individual level of the SEM represents the personal factors that influence behavior, including knowledge and skills. For instance, one way a behaviorist can promote allyship at the individual level is to review resources to increase knowledge and understanding to identify racist microaggressions, including in the workplace (Community Toolbox, 2020b). Microaggressions are “a subtle verbal or nonverbal behavior…directed at a member of a marginalized group, and has a harmful, derogatory effect” (Cunic, 2021). Active behaviors to address when a microaggression is given may serve as an intervention point for specific strategies addressed later in this paper.

Beyond the individual level, the interpersonal level of the SEM refers to social influence from friends, family, and colleagues, within social networks (McLeroy et al., 1988). For example, on an interpersonal level, behavior analysts can address racism by prioritizing diversity in supervision (e.g., through course material selection, clinical placements) while also connecting Black behavior analysts to other professionals to allow for a varied supervision experience. The community-level explores settings such as classrooms, workplaces, and neighborhoods, in which social relationships occur and seeks to promote changes within and across settings that may enable racism or racial equity. An example of active behaviors at the community level is to incorporate diverse representation in curricula and training materials to be more representative of a broader, diverse population. Or, to obtain and use information from those served regarding how to support culturally appropriate services and engage with supervisors to enhance services is another example.

Providing information and enhancing skills at the societal level looks at the broad societal factors that help create a climate in which racism is encouraged or inhibited. For example, behavior analysts can act as agents of change at the societal level by providing expert statements and testimonies to advocate for equitable delivery of services and/or access to treatment with populations served who may experience racism or discrimination (e.g., arrests of Black males with autism; insurance provider coverage of applied behavior analysis (ABA) treatment; insurance provider promotion of racial and ethnic providers). Behaviorists can model and engage in anti-racist behavior with our peers, colleagues, and those we serve through direct action (e.g., testimony, policy change) that actively opposes and challenges the structural systems in which individuals and groups continue to reinforce behaviors that are racist.

### Examining the ABCs of Racism

**Fig. 3:**
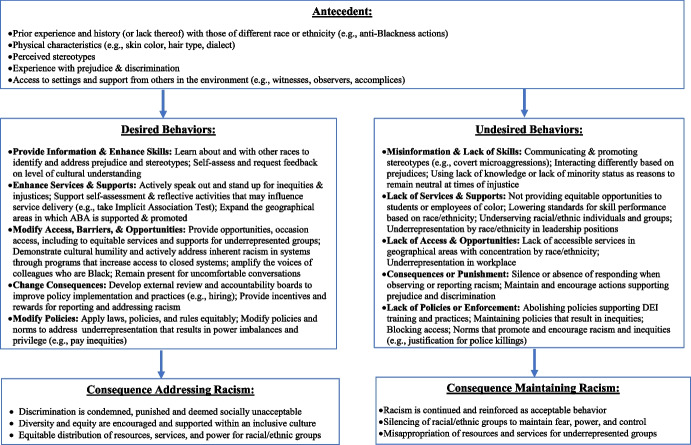
ABCs of systemic racism

In selecting the appropriate ecological level to address, it is important to consider the contextual factors (i.e., setting events), as well as what may serve as antecedents for engaging in the desired behavior and related consequence. Figure 3 illustrates how to begin to determine setting events that may influence the antecedent and consequence conditions. By examining the ABC (i.e., antecedents, behaviors, and consequences) related to racism, we are better positioned to consider a more comprehensive approach for addressing the problem. Identifying the setting events, including the different factors and broader contextual conditions present across ecological levels, may enhance our understanding of how to address racism, including both anti-Black and systemic racism. The SEM prompts consideration for a range of contextual factors (i.e., setting events) across ecological levels, which informs what may serve as antecedents for the desired behavioral response and related consequence. Figure 3 summarizes types of setting events at the individual, interpersonal, community, and societal levels.

For instance, a setting event at the individual level may include one’s behavioral history or experience including past traumatic events that may relate to the physical or biological characteristics of an individual (e.g., being detained by officers, followed by security, police called because not perceived as appropriate to be in a setting based on race). At the interpersonal level, a setting event may be a relational history of differential treatment by others who are of a certain race or ethnicity (e.g., interactions with peers). At the community or societal levels, prior history of discriminatory practices (e.g., hiring process, pay inequity) supported by cultural norms or policies may influence how one reacts or responds to various situations.

Antecedents are the stimuli (i.e., events, environmental conditions, experiences) that occasion operant responses. For instance, antecedents may include stimuli that evoke biases (e.g., favoring one group differently because of race), prejudices (e.g., perceptions that influence responding to those of a certain group based on race), and discrimination (e.g., unfair treatment based on race); or, may be events that impact access to settings (e.g., blocking of participation in workplace, neighborhoods, schools) or opportunities (e.g., detainment by police based on race). Determining the most appropriate active behavior to support requires situational analysis of the context (i.e., setting events) and antecedent conditions to then determine what is the most appropriate response (i.e., behavior) for the situation, to support an intended consequence (e.g., reduce racism). To better address racism using the ABC model, behaviorists should identify whose behaviors need to change across ecological levels and for various situations using a behavioral-community, culturo-behavior, or behavioral systems approach.

## Behavior Change Strategies Across Socio-Ecological Levels

Table 1 provides examples of how addressing systemic racism across ecological levels can be supported using a range of behavior change strategies. In addressing large-scale societal issues such as systemic racism, it is necessary to address the multiple and interrelated factors simultaneously through coordinated and comprehensive approaches within and across systems. In our roles as clinicians, supervisors, researchers, and educators, behaviorists must name and acknowledge active (e.g., providing opportunities for students of different races) or passive behaviors (e.g., not challenging other faculty who engage in racist behavior) that contribute to racism to effectively change behavior across the social-ecological levels. Generally, passive behaviors are avoidance or escape from doing an action.

The five categories of behavior change strategies consider both the antecedent and consequent conditions. The strategies support instructional (e.g., provide information and enhance skills), environmental (e.g., enhance services; modify access, barriers, and opportunities), or behavior management (e.g., change consequences and policy) techniques (Watson-Thompson et al., 2020). Antecedent interventions such as providing information to increase knowledge are important but are a weaker behavioral change as compared to stronger interventions that modify consequences (e.g., policy enforcement). In supporting comprehensive approaches, it is important to have a combination of strategies across ecological levels to modify the behavior of multiple individuals or groups. Additionally, how the strategies are implemented across ecological levels and by whom should be examined. Based on whose behavior is desired to be modified, behaviorists may serve as the targets (i.e., those whose behavior is targeted for change) and/or agents (i.e., those altering or managing contingencies that influence others’ behavior) of change depending on the ecological level most appropriate to address. Table 1 is not exhaustive; instead, it provides illustrative examples to comprehensively promote active behaviors to address racism within and across systems.

### Providing Information and Enhancing Skills

The first type of behavior change strategy listed in Table 1 is providing information and enhancing skills, which involves antecedent-based interventions that can be implemented across all levels of the social-ecological model. By actively engaging in personal learning and teaching, behavior analysts can act as both agents and targets of change. For example, at the individual level, an initial step for active behaviors to address both systemic and anti-Black racism is continuously learning the history and cultural context of Black people in the United States.

Behavior analysts are trained to understand the importance of learning histories. To address systemic racism, it is equally imperative to trace the historical roots of overt and operant racist behaviors of the past to the sometimes more covert and operant behaviors of the present. Thus, it is necessary to confront uncomfortable truths – truths that include looking at the prison and justice system as a modern-day form of oppression, Homeowner Associations (HOAs) as modern-day redlining, funding education through property taxes as modern-day segregation, and voter regulation efforts as modern-day voter suppression. Matsuda et al. (2020) examined the behavioral processes underlying expressions of racial bias and prejudice in humans and provide examples of behavior-analytic interventions that can help alter racist views, bias, and prejudice.

### Enhancing Services and Supports

Enhancing services and supports in addressing systemic racism refers to occasioning responding through changes in environmental variables and contingencies to support more equitable provision of services. Some actions can be taken across socio-ecological levels to set the conditions that support equitable services, particularly for and with Black individuals and groups. At the individual level, behavior analysts should identify personal biases that may influence service delivery through self-assessment and reflective activities. For instance, the Implicit Association Test (IAT) from Project Implicit and the Implicit Relational Assessment Procedure (IRAP) are resources for examining individual biases. At the interpersonal level, more empathetic services can be supported by seeking to better understand the experiences of those served who are Black. Additionally, more diverse services and service providers are also critical for ensuring culturally appropriate service delivery (Kim & Kang, 2018; Steinka-Fry et al., 2017). Occasioning the opportunity to actively listen to those from diverse backgrounds to better understand the history and experiences of both those served and also colleagues within the field is an important action.

There is a range of actions that can be supported by behaviorists to shift norms and practices that foster more equitable services and supports. For instance, at the interpersonal level when a colleague discusses the race of one child but not another, behaviorists then have an opportunity to engage with them as an agent of change and note the prejudice inherent in identifying (i.e., labeling) some individuals by race, while not others. Or, as an example at the community level, when consulting in schools where Black children are disproportionately restrained, secluded, suspended, or expelled, behaviorists need to be able to use their knowledge and skills to compel for the more equitable delivery of supports. At a broader societal level, behaviorists can be agents of change by advocating for the potential contributions of behaviorists in contributing to emergency response efforts across systems, including the schools and law enforcement (e.g., school crisis team inclusion of ABAs, behavior specialists assisting officers with crisis responding to de-escalate situations). The crisis intervention team model, for example, is evidence-based for officer-level outcomes such as changes in knowledge, attitudes, and decision-making for how to respond. Additional research is needed to examine the effects on officer-level behavior change and, ultimately, community or agency-level outcomes (e.g., arrests, detainment, police-involved injuries) (Watson et al., 2017), which may be an area that behaviorists can contribute to.

Community-level strategies such as ensuring equitable representation and inclusion in leadership and staffing structures also help to address racism in service delivery through more inclusive practices. Both collecting and openly reporting the demographics of professionals in the field will help to better assess our diversity regarding who is both delivering and receiving services and at what levels. As a field, more accurate collection and reporting of demographic data in both practice and research may permit a better understanding of who is (or is not) being served through behavior-analytic approaches to better identify gaps in service provision. Additionally, the generality of ABA practice and research findings for populations and settings can only be understood through demographic and geographic reporting requirements, which are an example of editorial policy and practices to support change.

Behavior analysts may act as agents of change in the community by examining and promoting the delivery of resources and services to individuals of diverse backgrounds and groups across demographic and geographical areas. Due to systemic racism, the underfunding and inequitable treatment often experienced in communities of color across settings and systems (e.g., school, law enforcement, healthcare, social service) inevitably lead to disproportionate services and other impacts that are long-lasting. For instance, within the school setting, schools serving primarily students of color received $23 billion less than schools serving primarily White students, despite similar population sizes in 2016 (Meatto, 2019). Also, research shows the criminalization of Black children in schools begins early and results in a school-to-prison pipeline (Bryan, 2020). The schools are a setting in which ABAs often work and may have opportunities to influence change. The underfunding and disproportionate access to resources, including ABA services and behavioral interventions for some populations and groups to address challenging behaviors is another example of inequities related to systemic racism. In general, there may be less access to behavior-analytic supports for students in under-resourced environments, which often overlap with communities with higher concentrations of Black people and other racial groups. An expansion in the range of areas in which behavior-analytic approaches are applied may promote enhanced use and consideration of our science to address a range of socially significant issues (e.g., racism, behavior skills, and discrimination/bias training for law enforcement).

### Modifying Access, Barriers, and Opportunities

Modifying access, barriers, and opportunities may contribute to more equitable conditions by facilitating change within the environments in which responding occurs. When determining if services are equitable within the community consider the following: Are reliable transportation or childcare common barriers? Are members of the community served represented in the workplace? Are all the voices at the table for decision-making and do they have a chance to be heard? Said another way, are Black people meaningfully and authentically included in decision-making processes? Is their input used and valued to guide actions and improvement? Use these questions to guide changes across settings, including in the work environment.

Behaviorists can help reduce barriers experienced by Black colleagues by amplifying Black voices to not only be heard and listened to but then facilitate action together as partners with Black colleagues to address underlying conditions. To be in accompaniment means to work together with Black colleagues to address the conditions that contribute to marginalization and then be committed and willing to share in the consequences of facilitating disruptive actions necessary for systemic change. In predominantly white spaces, this looks like making space or occasioning the opportunities for Black people to share and then modeling and supporting conditions to ensure that the dialogue is respectful and providing protected space for Black people to share and be supported in their actions without unduly adding to their burden. It is important that behaviorists are aware of their cognitive dissonance (i.e., the tendency to reduce psychological discomfort between two contradicting or “dissonant” cognitions by avoiding information likely to increase dissonance) (Festinger, 1957). Making space can involve accompanying Black individuals by working together in facilitating action and also sharing in the punitive or rewarding consequences.

When families of Black participants share their experiences with behaviorists, it is helpful to not rush to defend the science or practice, but first seek to understand the perspectives of different populations and groups, and then support facilitated action to address the concerns. For instance, when people have suffered and share their suffering and experiences, it is important to first listen as an ally and respond by demonstrating cultural humility and compassion rather than defensiveness and disdain, which will be further discussed later. When ABA practices are not culturally sensitive, to those served it may be considered racist, oppressive, or even coercive by service recipients and stakeholders. However, better assuring socially valid practices through feedback from constituents, including those underrepresented in services along with those partnering or providing services may assist in proactively addressing barriers to service access and delivery (Fawcett et al., 1980). Additionally, assuring conditions that support meaningful inclusion of Black professionals in delivering behavior-analytic services may also enhance access to underserved settings and populations.

It is critical to intentionally occasion access and opportunities for Black ABA professionals. For instance, White professionals can broker access to networks and also promote the success of Black colleagues through both formal and informal mentoring, networking, and support activities. As agents of change at the interpersonal level, behavior analysts can facilitate authentic opportunities for colleagues who are people of color to expand their networks. Mentorship by White to Black professionals that is culturally responsive can counter institutional barriers by providing access to networks and guidance for how to navigate within the unspoken culture of systems. Mentorship should be culturally competent (see texts like *Multiculturalism and Diversity in Behavior Analysis* [Conners & Capell, 2020] for a full definition of cultural competency in supervision) and ensure that supervisees are not required to adopt colonial practices. For instance, intentional mentorship can connect those who are Black with professional development opportunities (e.g., job opportunities, supervision) and resources (e.g., training, scholarships) that may be supported through more closed networks traditionally available to White individuals, but less accessible to racial and ethnic professionals. Additionally, promoting opportunities for Black behaviorists to connect with each other as a community of support is also critical so that there is a safe and supportive professional community, which in itself may redistribute power (e.g., Black Applied Behavior Analysts, Inc.; BABA). At the interpersonal or community levels, actions to support special interest groups, networks, and other forms of organizing that specifically provide support to and as identified by Black professionals should be reinforced. Consider with Black colleagues the most appropriate active behaviors (e.g., ally, accompany, advocate) to be supported based on the situation.

At the community level, compensating colleagues who are BIPOC for their equity work, as is done for other areas of expertise, can also demonstrate valuing the knowledge and experiences of Black individuals. To be clear, one person of an identity group cannot represent all people of the same identity. Neither should tokenism (e.g., checking the box for racial representation) be supported. However, recognition of specialized knowledge and experience, including related to race, ethnicity, and culture, should be openly discussed and rewarded based on the context and conditions. As an example, consider how to compensate Black colleagues for their professional services and emotional labor (e.g., paid time off work, monetary compensation, additional subsidized counseling and support service), instead of asking them to voluntarily expend their energy and reengage in traumatic experiences to advance the diversity goals of an organization. Said another way, do not rely on Black colleagues to provide additional unpaid labor in reforming the workplace or other settings in which underrepresentation may occur. However, it is important to provide opportunities and invite those who are Black to participate and engage, if they choose, in the ways self-determined to be most appropriate.

### Changing Consequences

Since behaviors are selected by their consequences, it is important to consider the controlling variables maintaining behaviors contributing to systemic racism. In changing consequences at the interpersonal level, one strategy is in responding to and disarming microaggressions when they occur. While the concept of microaggression was first used in 1970 by Chester M. Pierce for describing the daily degradation of the Black community by non-Black people in the United States (Sue, 2010), very scant progress has been made on systematic interventions to address it. More recently, Sue et al. (2019) introduced the concept of micro-interventions as an organizing framework of behavioral anti-racist strategies for combating everyday microaggressions across ecological levels. Analogous to the more widely known sexual assault bystander interventions framework, the concept of micro-interventions for daily racial microaggressions broadly refer to four strategies to be used by targets, allies, and bystanders. Steps to allow behaviorists to act as agents of change and span across the behavior change strategies include: (a) making the invisible visible by naming the oppressive act, condition, or process and thereby changing consequences; (b) disarming the microaggression by responding to the aggressor so that the intended function is disrupted (e.g., interrupt, protest, veto policies); (c) educate the offender and discuss the harmful effects of microaggressions; and, (d) seek external intervention, including support and reinforcement from others to further address the issue (Sue et al., 2019). Using micro-interventions when witnessing microaggressions can shift cultural practices and tolerance through anti-racist responses.

At the community level, behaviorists can determine how to change the consequences that may encourage or maintain systemic racism. For those in positions of influence, such as supervisors or faculty members, some strategies may include providing incentives to increase diversity in training, workplace, or service settings. Additionally, providing opportunities and protection for meaningful participation of Black community members to engage in providing feedback to support more equitable practices may also be helpful. Providing opportunities for feedback through mechanisms that are safe and protected may increase the likelihood that those from historically excluded racial and ethnic groups may continue to participate in culturally informed services and/or workplaces. When Black colleagues or clients do choose to share, do not minimize their experience, or defensively question the motive or intent of the offender (e.g., Are you sure that is what the person meant?), as that is a further microaggression and punisher, which contributes to and perpetuates the oppressive system.

At the broader systems level, leaders of organizations can examine the policies that shape the culture of the environment to contribute to addressing systemic racism. As noted in Table 1, evaluating and addressing pay structures that perpetuate economic disparities is critical. Additionally, modifying practices for salary negotiations that favor those with privilege can support implementing more equitable hiring and recruitment processes to systemically address pay inequities. Promoting transparency within a system through open pay structures, inclusive representation in leadership, and support for cultural and religious holiday inclusion or exclusion can demonstrate respect for those from traditionally marginalized groups. Behaviorists can utilize knowledge of motivation and generalized reinforcers to promote equity in policies, including in the workplace. Efforts to address socioeconomic determinants (e.g., education, employment, social connectedness) that often fuel systemic racism will contribute to a more equitable environment.

### Modifying Policy and Broader Systems

At the societal and broader systems level, behaviorists have an important role as advocates. **“**Americans have long been trained to see the deficiencies of people rather than policy. It is a pretty easy mistake to make: People are in our faces. Policies are distant” (Kendi, 2019, p. 28). To address systemic racism, modifying policies and broader systems is a key behavior change strategy that impacts other socio-ecological levels. At the individual level, a behaviorist may act as a target of change by examining workplace policies to identify procedures that do not promote equitable treatment of employees or assure equitable participation from all voices. As an agent of change, a behavior analyst may communicate with their direct supervisor about the need for changes to workplace policies. Behavior analysts can act as agents of change by establishing policies to support diversity-related continuing education and training for all staff, particularly for those who may not self-select to learn more about diversity, equity, and inclusion. Systems must continue to assure implementation of policies and practices that standardize expectations for cultural and diversity training, despite changes in the political or organizational climate.

There is a range of policies at the community level, including within the workplace setting, that disproportionately target Black people. Policy shifts in the workplace could include changes to dress code, which may disproportionately impact Black people, often specifically related to hairstyles, or banned types of clothing/footwear. Additionally, examining the schedules of holidays that are given as paid days, which are often focused on more traditional mainstream holidays, as well as increasing transparency around wages can help to support a more inclusive environment. For instance, there are cultural holidays including Kwanza, Martin Luther King Jr. Day, and Juneteenth that are of particular meaning to the Black community. Additionally, there are religious observations that are supported within cultures that should also be considered when providing flexibility for what individuals identify as appropriate paid holidays. In examining the Black experience in the workplace some considerations may include: Are work and service duties evenly distributed? Are Black colleagues included in decision-making? Do Black colleagues disproportionately receive punitive action as compared to White colleagues? Are Black colleagues tokenized or asked to speak on behalf of all people in their community? What is the pay gap between Black behavior analysts and White behavior analysts? Racism thrives in obscurity; as long as these questions remain taboo, racism pervades and informs our working conditions (Jameel, 2019). Our working conditions become our clients’ and consumers’ learning conditions; the impact is far-reaching.

At the community level, behaviorists should examine the cultural norms (e.g., editorial practices) that determine research that is considered socially valid. The process by which research and practice are validated (e.g., community-engaged research with participants) may diminish or exclude research paramount for serving in and with Black communities. Examining what is considered socially valid in the field of applied behavior analysis (ABA) and shifting policies to promote research in diverse areas may also help enhance the application of ABA within the field and across disciplines. Behaviorists should challenge our field in both research and practice to continue to adapt and grow in ways that are anti-racist. This is an opportune time for the field to explicitly include anti-racist and cultural humility modules in requirements such as the Verified Course Sequence for Board Certified Behavior Analysts (BCBAs).

At the societal level, behavior analysts can also address limited access to services for underserved populations. Often, behavior analysts may provide expert opinions, testimonials, or support other advocacy efforts that can help to recognize and address inequities across systems. Whether advocating for the equitable delivery of services and treatment of populations served, such as promoting expanded provider coverage for underserved groups, behavior analysts have the opportunity to act as agents of change at the societal level.

### Using Multiple Behavior Change Strategies to Address Police Brutality

In the context of police brutality, the socio-ecological approach provides an example of ways to intervene to impact change across socio-ecological levels. As we examine the issue of police brutality, it is important to note that we are focusing on the specific issue of police violence against Black males considering the recent killings, including the death of George Floyd. When looking at the issue of violence against Black people committed by police officers, it is evident that the primary target of change is those committing the acts of violence – the police. A situational assessment should be conducted to identify the skill deficit of law enforcement when responding to determine the expanded behavioral repertoire that is needed to be addressed. Creating systemic change in law enforcement will require behaviorists and other stakeholders to join forces with those organizing and advocating for change. For example, there may be an opportunity for behaviorists to work in a multi-faceted approach such as through the Neighborhood Funders Group (www.nfg.org), which is not an exhaustive list but suggests some organizations and foundations committed to addressing justice.

We acknowledge the need for systemic change to prevent police-involved acts of violence against Black people. Behaviorists can contribute to examining the contextual factors or setting events that may be associated with officer-involved shootings and fatalities. The data indicate that Black people are three times more likely to be killed by police than any other racial group (Sinyangwe et al., 2020); therefore, it is critical to look at the environment in which this violence manifests. It was found that law enforcement conduct traffic stops more routinely and conduct searches more frequently for Black drivers as compared to White or Hispanic drivers (Pierson et al., 2020). Additionally, White officers were more likely to use a gun than were Black officers, particularly in predominantly Black neighborhoods (Peeples, 2020). It is imperative to consider the issue of white fragility when service providers (e.g., law enforcement, educators, applied behavior analysts) respond to situations, particularly when involving racial and ethnic individuals and groups. White fragility is defined as, “a state in which even a minimum amount of racial stress becomes intolerable, triggering a range of defensive moves. These moves include the outward display of emotions such as anger, fear, and guilt, and behaviors such as argumentation, silence, and leaving the stress-inducing situation” (DiAngelo, 2011).

As we consider the multiple behavior change strategies that can be deployed across ecological levels, behaviorists have an opportunity to work in a multi-faceted approach to offer methods such as behavioral assessment, functional analysis, or behavior skills training to help not only identify what may be maintaining behavior but to inform appropriate interventions in partnership with law enforcement and other sectors. For instance, behavioral skills training for police officers could include presenting data that underscores the inequitable application of the use of force against Black people and an explicit explanation of punitive consequences for the continued inappropriate use of force. Police officers could participate in training to help recognize and combat bias. Roleplays could be conducted with the explicit goal of teaching police officers to practice and use their de-escalation skills (which they often demonstrate when interacting with other populations) when interacting with Black people. Police officers should be continuously evaluated and provided feedback on their interactions with people across different communities, in which technology like bodycams could prove useful (Peeples, 2020).

Interventions with law enforcement should examine broader community factors (e.g., reporting of inappropriate officer behaviors to an external accountability board to minimize repercussions; praise rather than silencing for those who report injustice) to create social norms intolerable toward the killing of those who are Black by police officers. Also, addressing policy (e.g., police conduct) that determines whether violent behavior, including from law enforcement, is reinforced, or punished is another example of an appropriate strategy that could be informed by behaviorists (Peeples, 2020). Many individuals and groups are organizing to collectively call for change and behaviorists, as scientists of human behavior, should lend their voices and expertise across the continuum of active behaviors (i.e., ally, activist, accompany, advocate, anti-racist).

## Cultural Humility: A Marathon Not a Sprint

When considering how to address systemic racism across ecological levels, it is critical to strive to demonstrate cultural humility. In identifying appropriate responses for enhancing diversity across socio-ecological levels, it is key to strive to not only practice cultural competence, but also cultural humility. Wright (2019) offers a look at cultural humility through a behavioral lens, suggesting that the work of a behaviorist on a path to cultural humility is never complete as a goal is to always be improving through enhanced cultural consciousness.

Table 2 displays the following levels of the cultural competence framework: (a) cultural knowledge, (b) cultural awareness; (c) cultural sensitivity; and (d) cultural competence (Community Toolbox, 2020c). The standard cultural competence framework is expanded to also include cultural humility (e). The framework is a continuum and each level builds upon the prior level. Therefore, one cannot practice cultural humility without having also demonstrated cultural competence, knowledge, awareness, and sensitivity. The demonstration of cultural competence is the recognition that competence is a continuous process of growth, learning, and application for how to respond in ways that are culturally appropriate and support cultural humility.

**Table 2:** Cultural competency continuum

Type of Cultural Competence	Definition
Cultural Humility	Support a lifelong commitment to self-evaluation and self-critique; a commitment to fix power imbalances; developing partnerships with people and groups who advocate for others.
Cultural Competence	Capacity to respond appropriately to various cultural environments and support appropriate participation to enhance relationships for a mutual purpose.
Cultural Knowledge	Familiarizing oneself with the history, values, belief systems, and behaviors.
Cultural Awareness	Internal change in attitudes and norms supporting openness and flexibility.
Cultural Sensitivity	Knowing cultural differences and similarities exist without assigning values (for better or worse) to differences.

There is a growing body of literature to address cultivating culturally competent practices, including the recently published *Multiculturalism and Diversity in Behavior Analysis* (Conners & Capell, 2020). This critical text aligns with the Behavior Analysis Certification Board (BACB) code of ethics and highlights the areas in which the field fails to prepare clinicians. The chapters are set up to ease discussion, complete with case reviews and questions. The work connects to an article by Dr. Brodhead (Culture always matters, 2019), where he lays out a compelling case for a critical analysis of *The Professional and Ethical Compliance Code for Behavior Analysts*. Additionally, Fong and Tanaka (2013) identify cultural competency as a vital asset for behavior analysts’ in scientific, technological, and clinical skills. The science of human behavior can be pivotal in helping shift the paradigm, and it will be stronger when behaviorists work together to openly name the problem: racism (Matsuda et al., 2020), and then engage in active behaviors to address it.

### Cultural Sensitivity and Awareness

At the lowest level of the continuum is cultural sensitivity, or knowing cultural differences and similarities exist. If one practices cultural sensitivity, then they behave in ways that minimize stereotyping, particularly in regard to verbal behavior. The next level is cultural awareness, indicating there is a change in perceptions and attitudes toward people of different cultures. Training programs and educational activities facilitated to better understand different cultures, races, and ethnicities, perceptions, and attitudes toward different groups are supported. For instance, diversity training on microaggressions can enhance cultural awareness.

### Cultural Knowledge

Beyond cultural awareness is cultural knowledge, or familiarity with the history, values, belief systems, and behaviors. Often, through intentional exposure to those of different races and ethnicities, there may be a change in perceptions. For instance, through opportunities to be immersed in varying cultures or with individuals and groups of different races, a deeper understanding and awareness can develop. Often, racism, which is a social construct, can be based on the lack of familiarity and comfortability with those who are of a different race. Therefore, practices to support diversity are helpful and should be supported intentionally across settings (e.g., schools, workplaces, neighborhoods). As people develop cultural knowledge, individuals must be willing to counter preconceived notions that relate to prejudices.

### Cultural Competence

Cultural competence refers to the process by which individuals and systems respond respectfully and effectively to people of all cultures, languages, classes, races, ethnic backgrounds, religions, and other diversity factors in a manner that affirms and values the worth of individuals and groups and protects the dignity of each (National Association of Social Workers, 2008). Cultural competence is the ability to behave in ways that actively demonstrate respect for differences through cultural norms (i.e., values, practices, and customs) (Community Toolbox, 2020c). Individuals can engage as targets of change to learn cultural competence through a broadened experience (e.g., travel, time spent abroad), effective models (e.g., mentors, colleagues), and through feedback from others in the environment (e.g., friends, co-workers). Based on a systems perspective, Fong & Tanaka (2013) summarize, “cultural competence as a set of congruent behaviors, attitudes, and policies that come together in a system, agency or among professionals and enable that system, agency or those professions to work effectively in cross-cultural situations” (p. 18).

The term cultural competence may be misleading as “competency” suggests that it is a skill that can be mastered, which is not the case. Researchers have also found challenges with some cultural competency measures. For many competency measures, whiteness was understood and framed as the norm (Kumas-Tan et al., 2007). Cultural incompetence was often framed as due to a lack of knowledge about the “other.” The measures ignored power relations of social inequality and assumed individual knowledge and self-confidence are sufficient for change (Kumas-Tan et al., 2007). Cultural competence requires intentionality and a process of continuous improvement and reflection to ensure generality in responding in ways that are culturally competent for various situations (e.g., race-related stimuli). To be culturally competent requires ongoing reflection and practice for how to behave in ways that demonstrate the utmost respect for differences across individuals and groups. To better promote the idea that continuous self-reflection is required, in this paper the standard cultural competency framework was expanded to also include cultural humility.

### Cultural Humility

In Table 2, cultural humility is a level higher than cultural competence. Cultural humility is an iterative and continuous process of self-reflection and self-critique; whereby, one not only learns about another’s culture, but probes more deeply to examine their own beliefs and cultural identities (Yeager, 2013). Cultural humility is characterized by demonstrating a lack of superiority toward an individual’s cultural background and experience (Hook et al., 2013). Through cultural humility power and privilege are examined, which is critical for supporting any of the continua of active behaviors. Wellman (1977) suggests “the essential feature of racism is not hostility or misperception, but rather the defense of a system from which advantage is derived on the basis of race” (p. 210). Thus, cultural humility requires not only recognizing but also addressing the advantages or privileges received by an individual or group that serve as a function of racism in the environment in which one operates.

Cultural humility requires digging deep through self-assessment and trusted feedback to understand one’s history through an awareness of the legacies of oppression against certain groups of people, including Black people. As people work to address systemic racism through active behaviors, it is helpful to collectively and more accurately frame history regarding Black people and also other racial and ethnic groups. A balanced American history that accurately reflects the rich diversity and also sorrows that are interwoven, including that of the Black experience, is necessary, including in our field. In addition to more accurate understandings of the Black experience from pre- to post-slavery, the inclusion of the history of Black people in the United States that includes innovation, community, empowerment, and leadership is also necessary for supporting cultural humility. As Kendi (2020) noted in a TED talk:

The heartbeat of racism is denial, and really the heartbeat of anti-racism is confession; [it] is the recognition that to grow up in this society is to literally at some point in our lives probably internalize ideas that are racist. Ideas that suggest certain racial groups are better or worse than others.

It is important for behavior analysts to critically assess our level of progress and set goals for how to continue to move along the continuum toward supporting cultural humility. Additionally, it should be noted that in the iterative process, self-reflection is necessary regarding where one may be on the continuum for different races, ethnicities, and cultures. Thus, just because cultural humility is demonstrated for some individuals and groups does not mean the behaviors will generalize to other racial and ethnic groups without intentionality and continued development of the repertoire. To actively support active behaviors on the continuum from allyship to anti-racism, one should determine how to contribute to addressing systemic racism through cultural humility by which sustained changes can be fostered within and across systems.

## Conclusion

Throughout history, systemic racism has negatively impacted Black people in the United States. Although racial inequities have continued to persist, there is even greater awareness of the magnitude of the disparities largely due to media-facilitated public observation (e.g., videos of police brutality, media awareness of COVID-19 disparities). As with the Civil Rights Movement of the 1960s, the visual images (i.e., stimuli) of inequities and oppression toward Black people is for many non-Black people a continued awakening and stark realization of the historical and continued manifestation of systemic racism. The COVID-19 pandemic has further magnified the lethality of America’s systemic maltreatment and neglect of Black people (Morial, 2020). Due to COVID-19 restrictions, there were fewer competing stimuli to distract people from the realities of systemic racism apparent by disparities in mortality and life expectancy for Black people as compared to White people (Morial, 2020).

The current problems of COVID-19 and police brutality, which both disproportionately impact Black people, signifies the true underlying pandemic of systemic racism (Elizondo-Urrestarazu, 2020; Morial, 2020). Systemic racism is embedded across ecological levels and systems, which thereby requires comprehensive approaches to simultaneously move multiple systems and behaviors as targets and agents of change. The problem of systemic racism is exasperating, but not limited to law enforcement and the healthcare system. Systemic racism is interwoven across institutions and systems as a part of cultural norms and policies. The underlying factors or social determinants (e.g., employment, education, social connections, access to resources) that need to be interrupted across systems, including our own, are related to disparities in socioeconomic conditions that serve as consequences of systemic racism. Thus, the dismantling of systemic racism requires all systems, including the field of applied behavior analysis, to consider through practice and research how to contribute to a more equitable community and society.

Through the devastating loss of lives, the mobilization of concerned individuals and groups desiring to contribute to addressing systemic racism is paramount. From teen activists like those who began the Katy4Justice movement in Katy, Texas, to progressive District Attorneys, like Kim Gardner in St. Louis, Missouri, many are working to identify and implement strategies within their influence. Behavior analysts both individually and collectively have the same opportunity to consider how to contribute to dismantling systemic racism within our systems of influence across a continuum of active behaviors from allyship to anti-racism.

In applying behavior change strategies across multiple aspects of the environment, behavior analysis has the opportunity to support a comprehensive and integrative approach for addressing systemic racism. To create a better future and address the issues of our time, understanding the historical roots of racism and behaving to support a continuum of active behaviors across social-ecological levels helps to create the positive change so desperately needed. The purpose of this paper is to provide an illustrative menu of actions to be supported in addressing systemic racism by offering behavior change strategies across socio-ecological levels and the continuum of active behaviors for anti-racism.

While a vaccine will be developed for infectious diseases, including COVID-19, for Black people there is still no cure for systemic racism or anti-Blackness. Systemic racism as an applied problem of social significance commissions behaviorists to consider how to contribute to addressing the problem. The science of behavior is well-suited to examine the contingencies governing behaviors within and across systems, which is pivotal for addressing operant behaviors influencing long-term behavior change. Additionally, we need to continually assess our progress in making these long-term changes.

Behaviorists must make a meaningful change to stop the transmission of systemic racism by first intervening within our systems of control. The behavioral science community can continue to have conversations but should also utilize tools and take actions that demonstrably facilitate a more equitable environment. When environments promote racial equity and justice, everyone benefits, including those served. By acknowledging and addressing past and present inequities, behaviorists can continue to grow by tackling systemic racism as an applied problem worthy of our collective effort and commitment. In the words of Michelle Alexander (2010), *The New Jim Crow*:

Seeing race is not the problem. Refusing to care for the people we see is the problem. The fact that the meaning of race may evolve over time or lose much of its significance is hardly a reason to be struck blind. We should hope not for a colorblind society but instead for a world in which we can see each other fully, learn from each other, and do what we can to respond to each other with love. That was King’s dream— a society that is capable of seeing each of us, as we are, with love. That is a goal worth fighting for.

— Michelle Alexander, *The New Jim Crow*

## References

[CR1] Alexander, M. (2010). *The New Jim Crow: Mass incarceration in the age of colorblindness*. The New Press.

[CR2] American Public Health Association. (2020). *Racism and Health. *Retrieved on August 30, 2021, https://www.apha.org/topics-and-issues/health-equity/racism-and-health

[CR3] Ardila Sánchez, J. G., Cihon, T. M., Malott, M. E., Mattaini, M. A., Rakos, R. F., Rehfeldt, R. A., & Watson-Thompson, J. (2020). Collective editorial: Ten guidelines for strategic social action. *Behavior and Social Issues,**29*(1), 15–30. 38624429 10.1007/s42822-020-00038-8 PMC7682518

[CR4] Aspen Institute. (2016, July 11). 11 terms you should know to better understand structural racism. *Aspen Institute.* https://www.aspeninstitute.org/blog-posts/structural-racism-definition/

[CR5] Atcheson, S. (2021). Performative allyship is in your workplace. Here’s what to do. *Forbes.* Retrieved on August 30, 2021 from: https://www.forbes.com/sites/shereeatcheson/2021/02/10/performative-allyship-is-in-your-workplace-heres-what-to-do-about-it/?sh=7ae860465ba9

[CR6] Bogat, G. A., & Jason, L. A. (2000). Toward an integration of behaviorism and community psychology. In J. Rappaport & E. Seidman (Eds.), *Handbook of Community Psychology*. Springer. 10.1007/978-1-4615-4193-6_5

[CR7] Brodhead, M. T. (2019). Culture always matters: Some thoughts on Rosenberg and Schwartz. *Behavior Analysis in Practice, 12*(4), 826–830. 10.1007/s40617-019-00351-8 31976295 10.1007/s40617-019-00351-8 PMC6834805

[CR8] Bronfenbrenner, U. (1979). *The Ecology of Human Development*. Harvard University Press.

[CR9] Bryan, N. (2020). Shaking the bad boys: Troubling the criminalization of black boys’ childhood play, hegemonic white masculinity and femininity, and the school playground-to-prison pipeline. *Race Ethnicity and Education, 23*(5), 673–692. 10.1080/13613324.2018.1512483

[CR10] Centers for Disease Control and Prevention. (2020). The Social-ecological model: A framework for prevention.Retrieved on August 30, 2021 from https://www.cdc.gov/violenceprevention/publichealthissue/social-ecologicalmodel.html

[CR11] Community Toolbox (2020a). Chapter 27: Providing information and enhancing skills. Retrieved on August 30, 2021 fromhttps://ctb.ku.edu/en/table-of-contents/implement/provide-information-enhance-skills

[CR12] Community Toolbox (2020b). Chapter 27, Section 5: Learning to be an Ally for People from diverse groups and backgrounds*. *https://ctb.ku.edu/en/table-of-contents/culture/cultural-competence/be-an-ally/main

[CR13] Community Toolbox (2020c). Chapter 20: Section 7. building culturally competent organizations*. *Retrieved on August 30, 2021 from https://ctb.ku.edu/en/table-of-contents/culture/cultural-competence/culturally-competent-organizations/main

[CR14] Conners, B. M., & Capell, S. T. (2020). *Multiculturalism and diversity in applied behavior analysis* (1st ed.). Routledge.

[CR15] Cooper, J. O., Heron, T. E., & Heward, W. L. (2007). *Definition and characteristics of applied behavior analysis* (2nd ed.). Pearson.

[CR16] Cunic, A., (2020). What are microagressions. Verywell Mind. Retrieved on August 30, 2021 from https://www.verywellmind.com/what-are-microaggressions-4843519

[CR17] Dahlberg, L.L., Krug, E.G. (2006). Violence: A global public health problem. In: Krug E, Dahlberg LL, Mercy JA, Zwi AB, Lozano R (Eds) (pp. 1–21). World Report on Violence and Health. Geneva, Switzerland: World Health Organization.

[CR18] De Houwer, J. (2019). Implicit bias is behavior: A functional-cognitive perspective on implicit bias. *Perspectives on Psychological Science, 14*(5), 835–840. 10.1177/1745691619855638 31374177 10.1177/1745691619855638

[CR19] Derman-Sparks, L., & Phillips, C. B. (1997). *Teaching/learning anti-racism: A developmental approach*. Teachers College Press.

[CR20] DiAngelo, R. (2011). White fragility. *International Journal of Critical Pedagogy, 3*(3), 54–70.

[CR21] Dryden, O., & Nnorom, O. (2021). Time to dismantle systemic anti-Black racism in medicine in Canada. *Canadian Medical Association Journal**193*(2), E55–E57. 10.1503/cmaj.201579. 10.1503/cmaj.201579 PMC7773037 33431548

[CR22] Edwards, K. E. (2006). Aspiring social justice ally identity development: A conceptual model. *NASPA Journal, 43*(4), 39–60. 10.2202/1949-6605.1722

[CR23] Elias, M. J., Neigher, W. D., & Johnson-Hakim, S. (2015). Guiding principles and competencies for a community psychology practice. In V. C. Scott & S. M. Wolfe (Eds.), *Community Psychology: Foundations for Practice* (pp. 35–47). Sage. 10.4135/9781483398150

[CR24] Elizondo-Urrestarazu, J. (2020). The other pandemic: Systemic racism and its consequences. Retrieved on August 30, 2021 from: https://equineteurope.org/2020/the-other-pandemic-systemic-racism-and-its-consequences/

[CR25] El-Mekki, S. (2018). Educational justice: Which are you- an advocate, ally, or activist? *The Education Trust*. Retrieved on August 30, 2021 from: https://edtrust.org/the-equity-line/educational-justice-which-are-you-an-advocate-ally-or-activist/

[CR26] Fawcett, S. B., Mathews, R. M., & Fletcher, R. K. (1980). Some promising dimensions for behavioral community technology. *Journal of Applied Behavior Analysis, 13*(3), 505–518. 10.1901/jaba.1980.13-505 16795630 10.1901/jaba.1980.13-505 PMC1308154

[CR27] Feagin, J. R. (2006). *Systemic racism: A theory of oppression*. Routledge.

[CR28] Festinger, L. (1957). *A theory of cognitive dissonance*. Row, Peterson.

[CR29] Fong, E. H., & Tanaka, S. (2013). Multicultural alliance of behavior analysis standards for cultural competence in behavior analysis. *International Journal of Behavioral Consultation and Therapy, 8*(2), 17–19. 10.1037/h0100970

[CR30] Goldiamond, I. (1974). Toward a constructional approach to social problems: Ethical and constitutional issues raised by applied behavior analysis. *Behaviorism, 2*, 1–84. 11664410

[CR31] Harper Browne, C. & O’Connor, C. (2021). Social ecological model of racism & anti-racism. *Center for the Study of Social Policy. *Retrieved on April 1, 2022 from https://cssp.org/wp-content/uploads/2021/12/A-Social-Ecological-Model-of-Racism-Anti-Racism.pdf

[CR32] Hook, J. N., Davis, D. E., Owen, J., Worthington Jr., E. L., & Utsey, S. O. (2013). Cultural humility: Measuring openness to culturally diverse clients. *Journal of Counseling Psychology, 60*(3), 353. 10.1037/a0032595 23647387 10.1037/a0032595

[CR33] Iacobucci, G. (2020). Covid-19: Racism may be linked to ethnic minorities’ raised death risk. *British Medical Journal, 369*, m2421. 10.1136/bmj.m2421 32554546 10.1136/bmj.m2421

[CR34] Jameel, J. (2019, February 28). Workplace discrimination is illegal. But our data shows it’s still a huge problem. *Vox. *Retrieved on August 30, 2021 from https://www.vox.com/policy-and-politics/2019/2/28/18241973/workplace-discrimination-cpi-investigation-eeoc

[CR35] Kendi, I. X. (2019). *How to be an antiracist. One World*. Penguin Random House LLC.

[CR36] Kendi, I.X. (2020, May). The difference between being “not racist” and anti-racist [Video]. TED Conferences. Retrieved on August 30, 2021 from https://www.ted.com/talks/ibram_x_kendi_the_difference_between_being_not_racist_and_antiracist

[CR37] Kim, E., & Jang, M. (2018). The effects of client-counselor racial matching on therapeutic outcome. *Asia Pacific Education Review,**19*, 103–110. 10.1007/s12564-018-9518-9

[CR38] Kumas-Tan, Z., Beagan, B., Loppie, C., MacLeod, A., & Frank, B. (2007). Measures of cultural competence: Examining hidden assumptions. *Academic Medicine, 82*(6), 548–557. 17525538 10.1097/ACM.0b013e3180555a2d

[CR39] Layng, T. V. J., Andronis III, P. T., Codd, R. T., & Abdel-Jalil, A. (2021). *Nonlinear Contingency Analysis*. Taylor & Francis. Retrieved on August 30, 2022 from https://bookshelf.vitalsource.com/books/9781000466263

[CR40] Man, J. (2021). Overt racism vs. covert racism- Understand meaning and implications. *Diversity for Social Impact*. Retrieved on August 30, 2021 from: https://diversity.social/covert-overt-racism/

[CR41] Marchildon, J. (2020). How racism and discrimination prevent access to good health and well-being for all. Global Citizen. Retrieved on August 30, 2021 from https://www.globalcitizen.org/en/content/marginalization-and-its-impact-on-health/

[CR42] Matsuda, K., Garcia, Y., Catagnus, R., & Brandt, J. A. (2020). Can behavior analysis help us understand and reduce racism? A review of the current literature. *Behavior Analysis in Practice, 13*, 336–347. 10.1007/s40617-020-00411-4 32642393 10.1007/s40617-020-00411-4 PMC7314880

[CR43] McLeroy, K. R., Bibeau, D., Steckler, A., & Glanz, K. (1988). An ecological perspective on health promotion programs. *Health Education Quarterly, 15*, 351–377. 10.1177/109019818801500401 3068205 10.1177/109019818801500401

[CR44] Meatto, K. (2019). Still separate, still unequal: Teaching about school segregation and educational inequalit*y.* New York Times. Retrieved on August 30, 2021 from https://www.nytimes.com/2019/05/02/learning/lesson-plans/still-separate-still-unequal-teaching-about-school-segregation-and-educational-inequality.html

[CR45] Morial, M. (2020, August 25). State of Black America Unmasked. *National Urban League*. Washington, DC. Retrieved on August 30, 2021 from http://www.stateofblackamerica.org/

[CR46] National Association of Social Workers (NASW). (2008). *Code of ethics*. No Author.

[CR47] Pachter, L. M., & Coll, C. G. (2009). Racism and child health: A review of the literature and future directions. *Journal of Developmental and Behavioral Pediatrics, 30*(3), 255–263. 10.1097/DBP.0b013e3181a7ed5a 19525720 10.1097/DBP.0b013e3181a7ed5a PMC2794434

[CR48] Peeples, L. (2020). What the data say about police brutality and racial bias- and which reforms might work. *Nature, 583*, 22–24. 10.1038/d41586-020-01846-z 32601492 10.1038/d41586-020-01846-z

[CR49] Pierson, E., Simoiu, C., Overgoor, J., Corbett-Davies, S., Jenson, D., Shoemaker, A., Ramachandran, V., Barghouty, P., Phillips, C., Shroff, R., & Goel, D. (2020). A large-scale analysis of racial disparities in police stops across the United States. *Nature Human Behaviour, 4*(7), 736–745. 10.1038/s41562-020-0858-1 10.1038/s41562-020-0858-1 32367028

[CR50] Prather, C., Fuller, T. R., Marshall, K. J., & Jeffries IV, W. L. (2016). The impact of racism on the sexual and reproductive health of African American women. *Journal of Women’s Health, 25*(*7*), 664–67. Retrieved on August 30, 2021 from https://www.apha.org/topics-and-issues/health-equity/racism-and-health 10.1089/jwh.2015.5637 PMC4939479 27227533

[CR51] Sinyangwe, S., McKesson, D., & Elzie, J., (2020). *Police Violence Map*. Mapping Police Violence. Retrieved from https://mappingpoliceviolence.org/

[CR52] Steinka-Fry, K. T., Tanner-Smith, E. E., Dakof, G. A., & Henderson, C. (2017). Culturally sensitive substance use treatment for racial/ethnic minority youth: A meta-analytic review. *Journal of Substance Abuse Treatment,**75*, 22–37. 10.1016/j.jsat.2017.01.006 28237051 10.1016/j.jsat.2017.01.006

[CR53] Sue, D. W. (2010). *Microaggressions in everyday life: Race, gender, and sexual orientation*. John Wiley & Sons.

[CR54] Sue, D. W., Alsaidi, S., Awad, M. N., Glaeser, E., Calle, C. Z., & Mendez, N. (2019). Disarming racial microaggressions: Microintervention strategies for targets, White allies, and bystanders. *American Psychologist, 74*(1), 128 https://psycnet.apa.org/fulltext/2019-01033-011.pdf 30652905 10.1037/amp0000296

[CR55] Trent, M., Dooley, D. G., Dougé, J., & SECTION ON ADOLESCENT HEALTH, COUNCIL ON COMMUNITY PEDIATRICS, COMMITTEE ON ADOLESCENCE, Robert M. Cavanaugh, Amy E. Lacroix, Jonathon Fanburg, Maria H. Rahmandar, Laurie L. Hornberger, Marcie B. Schneider, Sophia Yen, Lance Alix Chilton, Andrea E. Green, Kimberley Jo Dilley, Juan Raul Gutierrez, James H. Duffee, Virginia A. Keane, Scott Daniel Krugman, Carla Dawn McKelvey, Julie Michelle Linton, Jacqueline Lee Nelson, Gerri Mattson, Cora C. Breuner, Elizabeth M. Alderman, Laura K. Grubb, Janet Lee, Makia E. Powers, Maria H. Rahmandar, Krishna K. Upadhya, Stephenie B. Wallace. (2019). The impact of racism on child and adolescent health. *Pediatrics, 144*(2), e20191765. 10.1542/peds.2019-1765 31358665 10.1542/peds.2019-1765

[CR56] University of California Irvine. (2020). Building a culture where black people thrive. [Anti-Blackness: A definition]. Retrieved on August 30, 2021 from https://inclusion.uci.edu/confronting-anti-black-racism/change-the-culture/

[CR57] Watson, A. C., Compton, M. T., & Draine, J. N. (2017). The crisis intervention team (CIT) model: An evidence-based policing practice? *Behavioral Sciences & the Law, 35*(5/6), 431–441. 10.1002/bsl.2304 28856706 10.1002/bsl.2304

[CR58] Watson-Thompson, J., Francisco, V. T., & Anderson-Carpenter, K. D. (2020). A behavioral-community approach to community health and development: Tools for collaborative action. In: Cihon, T.M., Mattaini, M.A. (eds) *Behavior Science Perspectives on Culture and Community. Behavior Analysis: Theory, Research, and Practice. *Springer, Cham. 10.1007/978-3-030-45421-0_14

[CR59] Wellman, D. (1977). *Portraits of white racism*. Cambridge University Press.

[CR60] WHO Commission on Social Determinants of Health, & World Health Organization. (2008). *Closing the gap in a generation: health equity through action on the social determinants of health: Commission on Social Determinants of Health final report*. World Health Organization.

[CR61] Wright, P. I. (2019). Cultural humility in the practice of applied behavior analysis. *Behavior Analysis in Practice, 12*(4), 805–809. 10.1007/s40617-019-00343-8 31976292 10.1007/s40617-019-00343-8 PMC6834807

[CR62] Yeager, K. A., & Bauer-Wu, S. (2013). Cultural humility: Essential foundation for clinical researchers. *Applied Nursing Research,**26*(4), 251–6. 10.1016/j.apnr.2013.06.008 23938129 10.1016/j.apnr.2013.06.008 PMC3834043

[CR63] Yulianto, V.I. (2020). We’ve been facing a pandemic of racism. How can we stop it? The Conversation. Retrieved on August 30, 2021 from https://theconversation.com/weve-been-facing-a-pandemic-of-racism-how-can-we-stop-it-140284

